# Liver Abscess Caused by Klebsiella Pneumoniae in the Absence of Hepatobiliary Disease

**DOI:** 10.7759/cureus.29789

**Published:** 2022-09-30

**Authors:** Saira Ibrahim, Naga Neelima Nallapaneni, Dharushana Muthulingam

**Affiliations:** 1 Internal Medicine, SSM Health St. Mary's Hospital, St. Louis, USA; 2 Infectious Disease, St. Louis ID PC, St. Louis, USA

**Keywords:** acute febrile illness, liver abscess aspiration, kleibsiella bacteremia, bacterial liver abscess, klebsiella liver abscess

## Abstract

*Klebsiella pneumoniae* is notorious for causing abscesses in patients without any previous hepatobiliary disease and is a cause of liver abscesses. We report the case of a 70-year-old man with a past medical history of hypertension and benign prostatic hyperplasia who presented with a cough and fever lasting two weeks. The findings from his physical examination were unremarkable, but his laboratory investigations were significant for elevated inflammatory markers. A computed tomography scan of his abdomen revealed a complex septated cyst in the right lobe of his liver, and his blood culture was positive for *K. pneumoniae*. He responded well to drainage and intravenous and oral antibiotics. His abscess resolved fully. Cases of *K. pneumoniae* in the United States are rare, and studies are necessary to explore its epidemiology. Atypical symptoms make diagnosis a challenge, and physicians are urged to retain high levels of suspicion to diagnose and treat such cases early.

## Introduction

*Klebsiella (K.) pneumoniae* is a gram-negative, lactose-fermenting, rod-shaped bacterium that is part of natural intestinal flora and one of the common causes of nosocomial infections [1]. The liver is one of the viscera in which abscesses frequently form. There are multiple risk factors that can predispose a person to abscess formation. A primary liver abscess is an abscess that occurs in the absence of intraabdominal or hepatobiliary disease and any abdominal surgery.

We present a case of a 70-year-old man, without the above-mentioned risk factors for abscess formation described above, who presented with fever and was found to have *K. pneumoniae* bacteremia in the presence of a complex septated abscess in the right lobe of his liver. Timely diagnosis is a challenging task but earlier diagnosis and effective treatment can minimize the sequelae.

## Case presentation

A 70-year-old, African American man with a past medical history of hypertension and benign prostatic hyperplasia presented with fever, cough, and malaise lasting two weeks. He was on an angiotensin-converting enzyme inhibitor and amlodipine for his hypertension. On admission to our hospital, he was febrile, with T-max 103.3°, and tachycardiac with a heart rate of 130s. The patient had no history of IV drug or alcohol use or recent history of travel.

The findings from his abdominal, cardiovascular (CVS), and pulmonary examination were unremarkable. His laboratory workup revealed his WBC count to be within the reference range, but he had elevated C-reactive protein and procalcitonin levels. His polymerase chain reaction test was negative for coronavirus disease 2019. Two consecutive blood cultures were positive for *K. pneumoniae* at 11.5 and 14 hours from his presentation. The patient had no history of bacteremia. The bacteria were sensitive to ceftriaxone, meropenem, and trimethoprim/sulfamethoxazole but resistant to ciprofloxacin. His urinalysis showed 11 white blood cells and 1+ proteins, and his urine culture was negative. His serum creatinine and liver function tests were satisfactory throughout his hospital stay.

**Figure 1:**
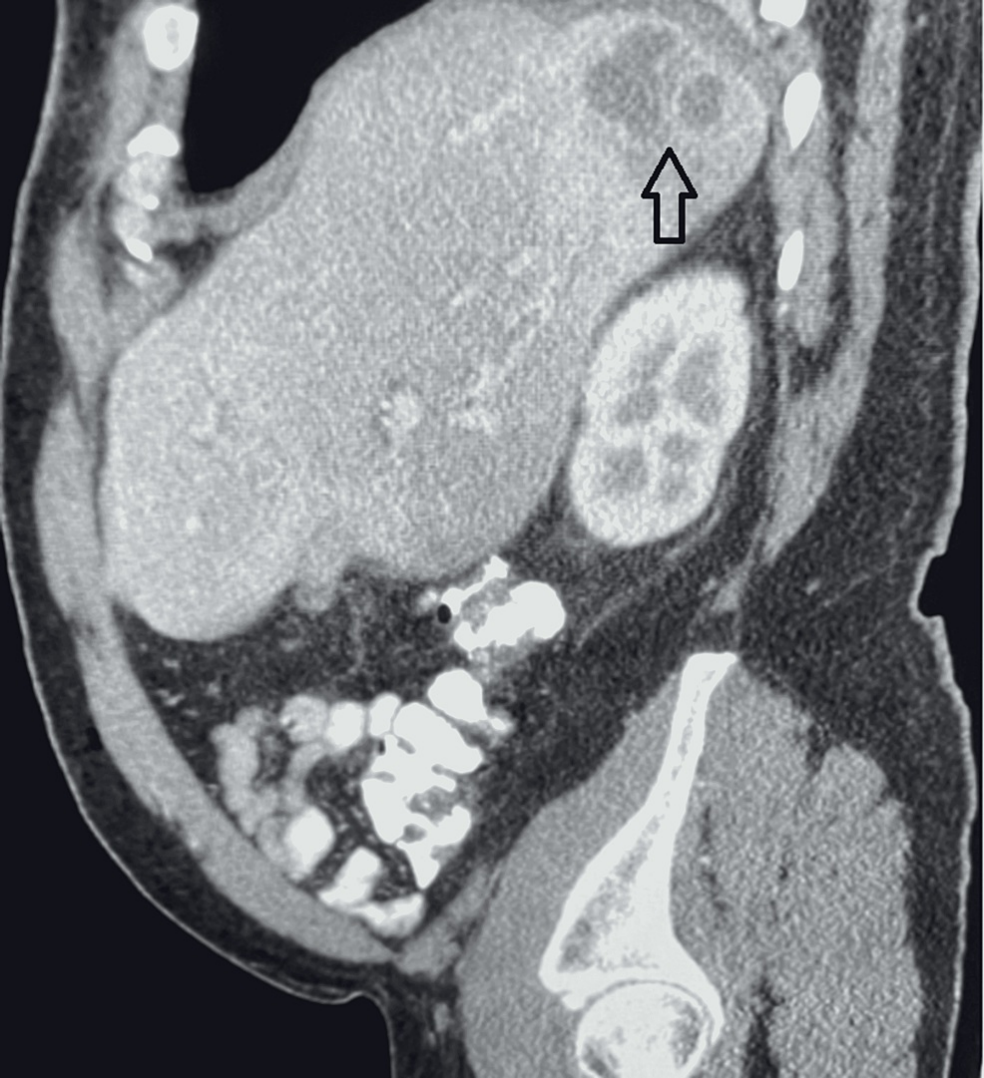
Sagittal abdominal computed tomography scan showing an abscess of the posterior segment in the right lobe of the liver (black arrow)

**Figure 2:**
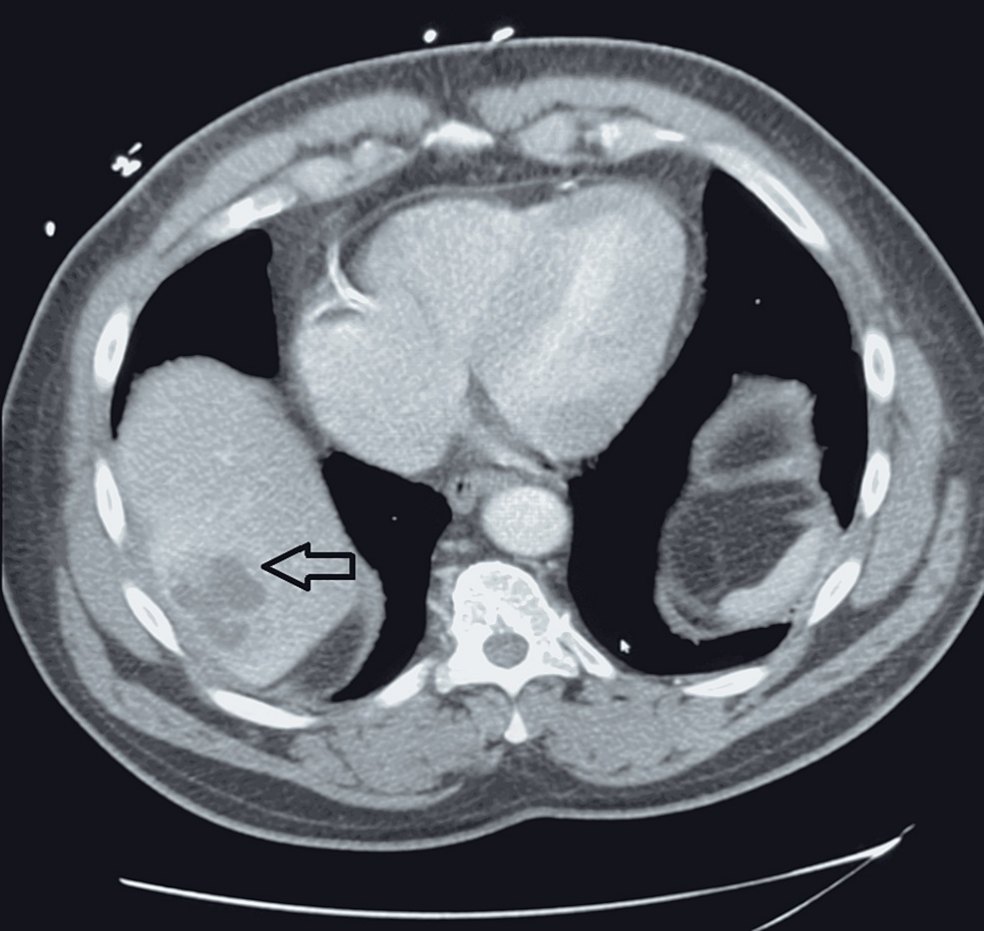
Transverse abdominal computed tomography scan showing a complex cystic area in the right lobe of the liver consistent with an abscess (black arrow)

His chest radiograph was normal, and we conducted a computed tomography (CT) scan of his abdomen, which revealed a complex cystic area involving the posterior segment of the right lobe of his liver near the dome of the diaphragm. The cystic area had a septated abscess measuring 5.2 cm (Figures 1, 2). The CT also showed questionable wall thickening of the sigmoid colon versus non-distention. Distal abdominal aortic aneurysm with involvement of the left common iliac artery was noted.

**Figure 3:**
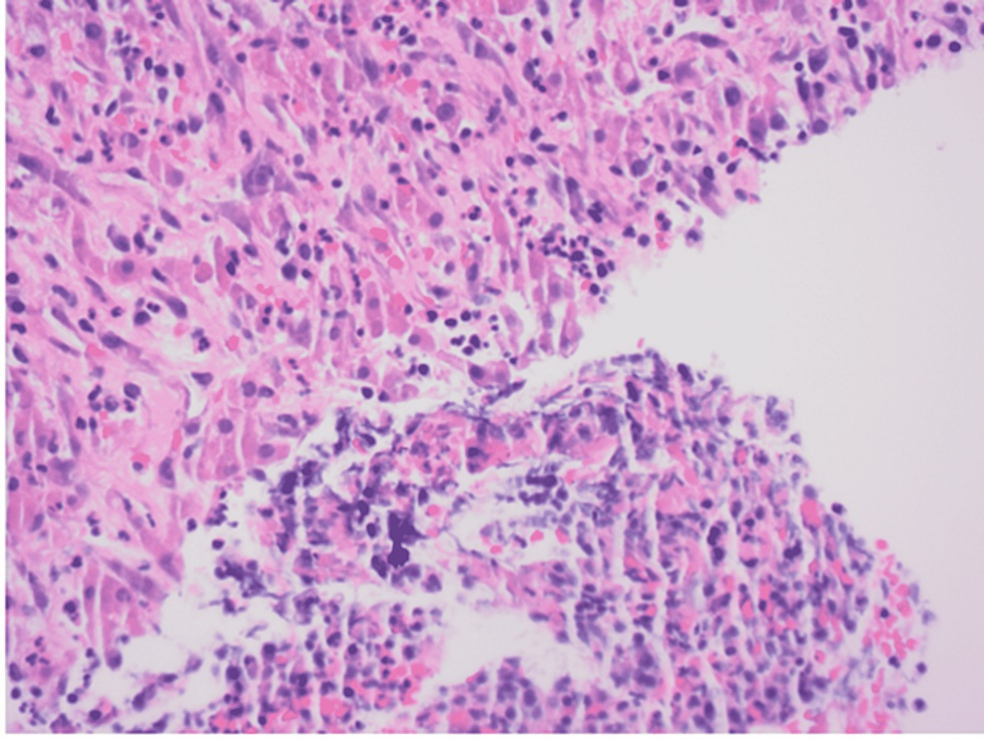
Histopathology of liver showing fibrinopurulent debris adherent to one core with neutrophils, infiltrating the hepatic plate of the adjacent edematous lobule consistent with an abscess

The interventional radiology team attempted aspiration of the abscess under imaging guidance but was unable to obtain any fluid. We sent four tissue biopsies of the liver for culture testing and pathology (Figure 3).

His liver biopsies were consistent with fibroblastic proliferation compatible with an abscess. His tissue culture also showed growth of *K. pneumoniae* sensitive to trimethoprim/sulfamethoxazole, meropenem, and ceftriaxone.

Due to the association of liver abscesses with colon cancer, we consulted with the gastroenterologist, and the patient underwent a colonoscopy. The colonoscopy revealed an 8-mm polyp in the transverse colon and a 20-mm polyp in the distal sigmoid colon; both were resected. Biopsies of the polyps showed tubular adenoma. The patient was treated with intravenous (IV) ceftriaxone and metronidazole in the hospital and was feeling better. He fully recovered, was afebrile, and subsequent blood cultures were negative. He was discharged home with trimethoprim/sulfamethoxazole and metronidazole twice daily for two more weeks. He was instructed to return for a repeat CT scan for a follow-up evaluation. During his follow-up evaluation at the infectious disease clinic, his CT revealed a 2.4-cm subcapsular lesion in the posterior segment of the right hepatic lobe representing a small hematoma at the previous biopsy site and no abscess.

## Discussion

*K. pneumoniae* has multiple serotypes, but the K1 capsular serotype and hypermucoviscosity phenotype are associated with community-acquired liver abscesses. These virulence factors also determine severe metastatic infections. The severe complications of *K. pneumoniae* infection are meningitis, endophthalmitis, septic emboli, and necrotizing fasciitis [1], but endophthalmitis is the most reported complication in the literature. However, serotyping was not done in our case, as it is not available in our facility.

Fever is the most common presenting symptom in a *K. pneumoniae* infection, followed by right quadrant pain. Other symptoms, such as anorexia, nausea, vomiting, and even right-sided pleural effusions, have been previously reported [2,3]. Data in the United States demonstrate very low mortality even with a short course of oral and IV antibiotics. Our patient received a short course of IV antibiotics followed by oral antibiotics, which cleared the abscess in six weeks.

In patients with *K. pneumoniae* infection, investigations often reveal leukocytosis, elevated erythrocyte sedimentation rate, and liver enzymes. Abdominal imaging is the investigation of choice for diagnosis and determining the patient's response to therapy. CT is preferred over ultrasound because the sensitivity of CT is almost 100%. A liver abscess should be considered in the differential diagnosis, and imaging is warranted in patients with *K. pneumoniae* bacteremia or refractory fever despite receiving appropriate antibiotic therapy [3].

Image-guided percutaneous drainage is the standard of care for diagnostic and therapeutic purposes [3]. If the abscess is immature and the pus collection is small, we can delay the drainage until the abscess matures or we can drain and leave the drain in situ. The size of the abscess only matters when choosing between percutaneous aspiration with a needle versus catheter placement. Medical treatment alone without drainage has been reported but limited data are available to compare. According to the current data, oral antibiotics are non-inferior to IV antibiotics in management [3]. The duration of antibiotics is four to six weeks [4].

Liver abscesses with *K. pneumoniae* are typically more prevalent in Asia than in Western countries, but the incidence is rising in Europe and the US [5]. Ninety-three cases were reviewed in a study, of which 39 were Asian patients living in the United States [6].

The prognosis of a *K. pneumoniae* infection is good, and the mortality rate of *K. pneumoniae *infections has significantly decreased due to timely detection and treatment [6]. The mortality rate with an abscess alone is high (5%) but doubles or triples with a concomitant bacterial infection [7]. Liver abscesses are associated with colon cancer [7], but villous adenomas can also cause mucosal disruption similar to colon cancer [8]. Colonoscopy should also be considered in patients admitted with an abscess.

In our case, the patient was treated with aspiration followed by oral antibiotics, and he recovered without any complications. CT also showed questionable wall thickening of the sigmoid colon versus non-distention, and due to the association of *K. pneumoniae* with colorectal malignancy, colonoscopy was done on an inpatient basis, and that was unremarkable.

## Conclusions

A primary liver abscess is an abscess that develops in the absence of any hepatobiliary disease and is not very common. Physicians should consider it as a differential for Klebsiella bacteremia, as if left undiagnosed, it can lead to significant morbidity. Our patient presented with high-grade fever and was bacteremic with* K. pneumoniae;* his urine culture and chest X-ray were normal. His persistent fever and bacteremia prompted the abdominal imaging, leading to timely diagnosis and treatment.

## References

[1] Jun JB (2018). Klebsiella pneumoniae liver abscess. Infect Chemother.

[2] Kamal F, Williams G, Akbar H, Khan MA, Kadaria D (2017). Klebsiella pneumoniae liver abscess: a case report and review of literature. Cureus.

[3] Molton JS, Chan M, Kalimuddin S (2020). Oral vs intravenous antibiotics for patients with Klebsiella pneumoniae liver abscess: a randomized, controlled noninferiority study. Clin Infect Dis.

[4] Moore R, O'Shea D, Geoghegan T, Mallon PW, Sheehan G (2013). Community-acquired Klebsiella pneumoniae liver abscess: an emerging infection in Ireland and Europe. Infection.

[5] Fazili T, Sharngoe C, Endy T, Kiska D, Javaid W, Polhemus M (2016). Klebsiella pneumoniae liver abscess: an emerging disease. Am J Med Sci.

[6] Rahimian J, Wilson T, Oram V, Holzman RS (2004). Pyogenic liver abscess: recent trends in etiology and mortality. Clin Infect Dis.

[7] Lai HC, Chan CY, Peng CY, Chen CB, Huang WH (2006). Pyogenic liver abscess associated with large colonic tubulovillous adenoma. World J Gastroenterol.

[8] Huang WK, Chang JW, See LC (2012). Higher rate of colorectal cancer among patients with pyogenic liver abscess with Klebsiella pneumoniae than those without: an 11-year follow-up study. Colorectal Dis.

